# A Case of Valproate Induced Hyperammonemic Encephalopathy

**DOI:** 10.1155/2011/969505

**Published:** 2011-05-04

**Authors:** Surjit Tarafdar, Mark Slee, Faisal Ameer, Matt Doogue

**Affiliations:** ^1^Nephrology, Flinders Medical Centre, Bedford Park, SA 5042, Australia; ^2^Neurology, Flinders Medical Centre, Bedford Park, SA 5042, Australia; ^3^General Medicine, John Hunter Hospital, Newcastle, NSW 2305, Australia; ^4^Endocrinology/Clinical Pharmacology, Flinders Medical Centre, Bedford Park, SA 5042, Australia

## Abstract

A 36-years-old man on phenytoin, levetiracetam, and sodium valproate presented with acute confusion. Routine investigations including serum valproate and phenytoin concentration were normal. His serum ammonia concentration was raised. His valproate was held and 2 days later he recovered with concordant normalisation of serum ammonia concentration. Urea acid cycle disorder was ruled out, and a diagnosis of valproate induced hyperammonemic encephalopathy (VHE) was made. Asymptomatic hyperammonemia occurs in 15–50% of valproate-treated patients, and while the true incidence of VHE is not known, it is a recognized complication of sodium valproate treatment. VHE typically presents acutely with impaired consciousness, lethargy, and vomiting. Valproate concentrations may be in the therapeutic range, and liver function tests are typically “normal.” Treatment for VHE consists of ceasing valproate and providing supportive care. Some have advocated carnitine replacement.

Patients with valproate induced hyperammonemic Encephalopathy (VHE) may present with impaired consciousness, lethargy or increased seizure frequency. These patients may have normal liver function tests and plasma valproate concentration leading to the attending physician overlooking the diagnosis of VHE. 

A 36-year-old man presented with acute confusion about 20 hours after an alcohol binge. Although he had long standing partial epilepsy, his partner had not observed any seizures and said this was unlike usual post ictal state. His regular medications were phenytoin 300 mg twice daily, levetiracetam 500 mg twice daily, and sodium valproate 1.5 g mane 2 g NOCTE. He had splenectomy 3 years previously for idiopathic thrombocytopenia with normal platelet counts since then. He was a current smoker and an alcohol binge drinker once or twice a month.

Findings at admission included normal physical examination, blood count, electrolytes, liver function tests, CRP, urinalysis, chest radiograph, ECG and head CT scan. Plasma ethanol  <  0.01 g/dL excluded acute alcohol intoxication and urine toxicology screen was negative. Plasma valproate was 52.9 mg/L (range 50–100 mg/L) and plasma phenytoin 13.8 mg/L (range 10–20 mg/L). Serum ammonia was high—284 umol/L (<50 umol/L). Valproate was withheld, his symptoms resolved over 24 hours, and on day 3 the serum ammonia was 36 umol/L. Screening for urea cycle disorders was negative. A diagnosis of valproate induced hyperammonemic encephalopathy (VHE) was made. 

The typical presentation of VHE is impaired consciousness and lethargy. Focal neurological symptoms and increased seizure frequency may be present [1]. The incidence of VHE is not known, but asymptomatic increases in serum ammonia are seen in 16%–52% of patients receiving valproate therapy [2]. VHE can occur with normal liver function tests and plasma valproate concentration [3]. 

Ammonia is eliminated by the urea cycle whose first rate-limiting step is mediated by carbamylphosphate synthetase1 (CPS1). CPS1 in turn is activated by N-acetylglutamate. 

The metabolism of valproate by mitochondrial oxidation produces propionyl Co-A and valproyl Co-A, which inhibit N-acetylglutamate synthetase with consequent depletion of N-acetylglutamate. This leads to the inhibition of CPS1 resulting in decreased clearance of ammonia [4, 5]. Another mechanism thought to play a role is the reduction of hepatic carnitine levels by valproate. Deficiency of carnitine results in decreased beta-oxidation of fatty acids, which in turn results in reduced levels of acetyl Co-A. Acetyl Co-A is a substrate for the N-acetylglutamate synthetase mentioned above. Thus the decrease in acetyl Co-A ultimately disrupts the urea cycle resulting in ammonia accumulation [6]. 

Treatment for VHE includes supportive care and withholding valproate. L-carnitine replacement is sometimes used [7, 8]. Extremely high concentrations of ammonia may warrant dialysis [9]. The diagnosis of VHE may be overlooked when the serum valproate concentration and liver function tests are within expected ranges. Physicians should be alert to this potential complication of valproate and measure serum ammonia in patients with alterations in mental status or unexplained increase in seizure frequency. Adjustment of dose or withdrawal of valproate may be necessary in the symptomatic patient in whom altered mental status is not due to seizure activity.
